# Prolonged warm ischemia time increases mitogen-activated protein kinase activity and decreases perfusate cytokine levels in *ex vivo* rat liver machine perfusion

**DOI:** 10.3389/frtra.2023.1215182

**Published:** 2023-08-25

**Authors:** Joohyun Kim, Seung-Keun Hong, Yongqiang Yang, Alice Lee, Karin M. Hoffmeister, Benjamin N. Gantner, Jong-In Park

**Affiliations:** ^1^ Department of Surgery, Division of Transplant Surgery, Medical College of Wisconsin, Milwaukee, WI, United States; ^2^ Versiti Translational Glycomics Center, Blood Research Institute and Medical College of Wisconsin, Milwaukee, WI, United States; ^3^ Department of Medicine, Division of Endocrinology, Medical College of Wisconsin, Milwaukee, WI, United States; ^4^ Department of Biochemistry, Medical College of Wisconsin, Milwaukee, WI, United States

**Keywords:** liver viability, warm ischemia time, cytokine, chemokine, machine perfusion

## Abstract

**Introduction:**

Machine perfusion is increasingly being utilized in liver transplantation in lieu of traditional cold static organ preservation. Nevertheless, better understanding of the molecular mechanisms underlying the ischemia-reperfusion injury (IRI) during *ex vivo* perfusion is necessary to improve the viability of liver grafts after transplantation using machine perfusion technology. Since key cellular signaling pathways involved in hepatic IRI may allow a chance for designing a promising approach to improve the clinical outcomes from this technology, we determined how warm ischemia time (WIT) during procurement affects the activity of mitogen-activated protein kinase (MAPK) and perfusate concentration of cytokines in an *ex vivo* rat liver machine perfusion model.

**Methods:**

Male Sprague-Dawley rats underwent *in situ* hepatic ischemia with varying WIT (0, 10, 20, 30 min, *n* = 5 each), and subsequently 3 h of cold ischemia time and 2 h of machine perfusion prior to determining the degree of MAPK activation-phosphorylation and cytokine concentration in liver tissue and perfusates, respectively.

**Results:**

Our data revealed a strong correlation between incremental WIT and a series of liver injury markers, and that prolonged WIT increases ERK1/2 and p54 JNK phosphorylation during machine perfusion. Notably, specific cytokine levels (MCP-1, MIP-2, GRO/KC, IL-10, and IL-5) were inversely correlated with the phosphorylation levels of ERK1/2, p38 MAPK, and p46/p54 JNK.

**Discussion:**

These results suggest that MAPK activation, specifically ERK1/2 and p54 JNK phosphorylation, have potential as a biomarker for hepatic IRI pathophysiology during machine perfusion. Elucidation of their functional significance may lead to designing a novel strategy to increase the clinical benefit of machine perfusion.

## Introduction

Currently, the only definitive treatment for end-stage liver disease is liver transplantation (LT). However, the shortage of available organs remains a major challenge in this field (1). A potential solution to this problem may stem from the use of marginal grafts which are typically discarded due to their poor tolerance to hepatic ischemia-reperfusion injury (IRI) (2). Machine perfusion systems have been introduced to mitigate IRI in marginal livers, especially those from donation after circulatory death, which shows an increased degree of injury under the increased warm ischemia time (WIT) during the organ donation process (3). Nevertheless, there is still limited understanding of the underlying pathophysiology of IRI during machine perfusion for LT, which hinders the development of diagnostic or therapeutic methods required for improving the viability of livers for LT (4). Therefore, it is critical to identify a molecular mechanism underlying the hepatic IRI. Identifying the cellular signaling pathways involved in the injury may lead to a promising strategy to improve patient outcomes after LT.

Mitogen-activated protein kinases (MAPKs) play crucial roles in responding to various stress signals, including hypoxia, and subsequent regulation of cellular responses critical to survival, proliferation, growth, migration, differentiation, and metabolism (5–7). Among the 14 mammalian MAPKs, extracellular signal-regulated kinase (ERK) 1/2, p38 MAPK, and c-Jun N-terminal kinase (JNK) have been hypothesized as key regulators of hepatic IRI given the strong correlation between their activity and the injury (5). In support of this notion, a recent study of human livers during normothermic machine perfusion has demonstrated that ERK1/2 activity increases in livers with prolonged cold ischemia time (CIT) (8), suggesting involvement of the kinases in the pathophysiology of IRI. Because different MAPKs have distinct roles (5), it is possible that certain MAPKs support liver cell survival from IRI while others contribute to the liver damage (9).

The dual nature of cell signals during hepatic IRI, which can be both injurious and reparative, has been attributed to the function of innate immunity (10). In this context, MAPKs are involved in regulating cytokine production during innate immune responses (11, 12). MAPKs regulate diverse cytokine responses, including both pro-inflammatory (13–17) and anti-inflammatory cytokines (18). In hepatic IRI, Kupffer cells initiate the production and release of cytokines (10). Kupffer cells release tissue necrosis factor alpha (TNF-α), which induces the expression of adhesion molecules, facilitating the adhesion of circulating neutrophils to the endothelial cells, resulting in cell extravasation (19, 20). TNF-α and interleukin-1 (IL-1) released by activated Kupffer cells also upregulate the expression of adhesion proteins on the surface of neutrophils and promote chemotaxis (21). TNF-α derived from Kupffer cells can induce chemokines of hepatocytes, mobilizing neutrophils towards injured areas (22).

Prior research has primarily concentrated on the role of cytokines on *in situ* IRI, rather than *ex vivo* machine perfusion (23–25). Exposure of the liver to ischemia under *ex vivo* conditions may develop different cellular responses. During *ex vivo* machine perfusion, decreased numbers of Kupffer cells (26–28) and neutrophils (29) may alter the immune response associated with cytokine release in the liver. A recent study on discarded human livers highlighted the increase in cytokine levels during normothermic machine perfusion, although their significance under the condition remains unclear (29). Therefore, investigating MAPK activation and cytokine production in response to incremental liver damage during *ex vivo* perfusion may provide insights into the involvement of the MAPK pathways in machine perfusion. This study determined the correlation between WIT, MAPK phosphorylation, and cytokine levels in an *ex vivo* rat liver machine perfusion model.

## Materials and methods

### Animals

Male Sprague-Dawley rats were housed in an animal facility accredited by the Association for Assessment and Accreditation of Laboratory Animal Care. They had *ad libitum* access to Purina Lab Diet 5,001 and reverse osmosis water. Surgical procedures were conducted under isoflurane inhalation anesthesia (1%–3%; oxygen flow 1 L/min). The rats were placed on a far infrared warming pad (Kent Scientific, Torrington, CT) set to maintain a temperature of approximately 39°C. All animals received humane care in accordance with the National Institutes of Health Guide for the Care and Use of Laboratory Animals. The Institutional Animal Care and Use Committee at the Medical College of Wisconsin approved all experiments (animal use application number 00004857).

### Total hepatectomy for *ex vivo* perfusion model

Male rats (median age of 8.5 weeks and median body weight of 330 g, interquartile range of 8.0–9.2 weeks and 310–363 g, respectively, Charles River Laboratories) were used for this study. There were four experimental groups (*n* = 5 each) based on WIT: 0 min, 10 min, 20 min, and 30 min. The procedure involved opening the abdomen with a cruciate incision, mobilizing the whole liver by incising ligaments, and ligating and dividing the phrenic vein and the right adrenal vein using 7-0 silk ties. Heparin (100 units, Sagent Pharmaceuticals) was injected into the intrahepatic inferior vena cava (IVC). Subsequently, the hepatic artery and the portal vein were dissected and ligated. *In situ* WIT was started, and the abdomen was temporarily closed using 2-0 nylon continuous stitches. After the designated WIT, the abdomen was reopened, and the portal vein was cannulated with a 16-gauge catheter while the infrahepatic IVC was ligated. The chest wall was opened, and the suprahepatic IVC was partially incised to exsanguinate the rat. The liver was flushed with 50 ml of cold lactated Ringer's solution with heparin (50 units) through the portal vein, and the portal vein cannula was secured in place. The suprahepatic IVC was cannulated using a 5 mm segment of polyethylene tubing (internal diameter 0.2794 mm and outer diameter 0.6096 mm, PE10, Braintree Scientific, Inc.). The whole rat liver was dissected and removed from the body. The liver was weighed and submerged in cold lactated Ringer's solution in ice for 3 h until it was connected to the normothermic machine perfusion system.

### Normothermic machine perfusion

The normothermic machine perfusion system was primed with 100 ml of perfusate (Table 1) in a glass reservoir, which was placed on a magnetic stirrer (Corning Pyroceram Top Digital Stirrer, Fischer Scientific) maintaining a temperature of approximately 38°C using a heated recirculator (Model 210, PolyScience). The system was connected to a peristaltic pump (Masterflex® Ismatec® Reglo, Avantor) and the oxygenator (fiber/membrane oxygenator, Harvard Apparatus) with a flow rate of 0.5 L/min using a mixture of 95% oxygen and 5% carbon dioxide. Previous studies have shown that red blood cells from different species can be used as oxygen carriers in rat liver machine perfusion experiments (30). To avoid the use of up to 100 rats for whole blood collection in this study, porcine red blood cells were utilized as oxygen carriers in the perfusate. Fresh pig blood was obtained from a local farm (Wilson's Prairie View Farm, Inc., Elkhorn, WI) and heparin (10,000 U/ml, Sagent Pharmaceuticals) and antibiotics (Penicillin-Streptomycin 10,000 IU/ml, ThermoFisher Scientific) were added before keeping the blood on ice. The whole blood was then filtered using sterilized gauze and centrifuged at 1,500 g for 5 min. After removing the supernatant, the pellet was filtered using a leukocyte filter (LeukoGuard® BC2 cardioplegia filter, Pall Corporation), and stored in a blood container (Transfer-pack, Fenwal Inc.) at 4°C for up to 2 weeks. The complete list of perfusate contents is summarized in Table 1.

**Table 1:** Perfusate component for normothermic machine perfusion.

Reagents	Amount in 100 ml	Manufacturer
Dulbecco's modified eagle's medium	72.3 ml	ThermoFisher scientific
Human albumin (Albutein, 4%)	3.2 ml	Albutein, Grifols
Human insulin aspart (100 IU/ml)	2 ml	Novo Nordisk
Penicillin-streptomycin (10,000 U/ml)	1 ml	ThermoFisher scientific
L-glutamine (200 mm)	1 ml	ThermoFisher scientific
Porcine packed red blood cell	20 ml	N/A
Heparin (1,000 U/ml)	0.5 ml	Sagent pharmaceuticals
Taurocholic acid sodium salt hydrate	25 mg	T4009-1G, Sigma-Aldrich

After the designated WIT and CIT, the rat liver was placed in a heated organ moist chamber (Harvard Apparatus) in the normothermic machine perfusion system. The portal vein was connected to the inflow metal tube connector, and the suprahepatic IVC was connected to the outflow metal tube connector. Polyethylene tubing placed during total hepatectomy was used to connect the blood vessels and metal connectors. The initial flow rate was set at 5 ml/min and was increased by 5 ml/min every 5 min up to a maximum of 30 ml/min or 12 mmHg of portal venous pressure, as measured by a monitor (PM-4 perfusion pressure monitor, Living Systems Instrumentation). After 2 h of machine perfusion, both perfusate and liver tissue samples were harvested. Approximately 0.5 ml of perfusate was retrieved from the glass reservoir situated above the magnetic stirrer, which was then centrifuged at 3,000 rpm for 10 min at room temperature to isolate the supernatant. These collected serum samples were promptly frozen at −20°C and subsequently stored at −80°C until further analysis.

### Immunoblot analysis

Proteins were extracted from snap frozen liver samples and quantified using the BCA reagent (Pierce, 23,225) (31). For SDS-PAGE analysis, 50 μg of protein was resolved and transferred to a polyvinylidene difluoride membrane filter (Bio-Rad, 162-0177). Membrane filters were then blocked in 0.1 M Tris (pH 7.5)-0.9% NaCl-0.05% Tween 20 with 5% nonfat dry milk and incubated with the following primary antibodies: ERK1/2 (Cell Signaling, 4,695, 1:2,000), phospho-ERK1/2 (Thr202/Tyr204, Cell Signaling, 4,370, 1:2,000), SAPK/JNK (Cell Signaling, 9,252, 1:2,000), phospho-SAPK/JNK (Thr183/Tyr185, Cell Signaling, 46,682, 1:2,000), p38 MAPK (Cell Signaling, 8,690, 1:2,000), phospho-p38 MAPK (Thr180/Tyr182, Cell Signaling, 4,511, 1:2,000), and glyceraldehyde-3-phosphate dehydrogenase (GAPDH, Cell Signaling, 2,118, 1:5,000). Detection of the signal was performed using the SuperSignal West Femto chemiluminescence kit (Pierce) and the ChemiDoc MP imaging system (Bio-Rad). Following the detection of phosphorylated protein forms, the same blot was stripped and reprobed for GAPDH, serving as a loading control, as well as the total MAPK proteins. We quantified protein phosphorylation as a ratio of phosphorylated to total protein expression, using densitometry with Image Lab 6.1 software (Bio-Rad, Hercules, CA). Expression levels within each WIT group were normalized against those in the 0 min WIT group, which served as the control for the standardization of densitometry data (Supplementary Figure S1). Specifically, we normalized the expression levels of phosphorylated MAPKs against the optical density (O.D.) of the 0 min WIT group. This involved dividing the O.D. of the phosphorylated protein by the O.D. of the total protein, and subsequently normalizing this ratio using the mean value of the control group. As a result, the expressions at WIT 10, 20, and 30 min are presented as relative expressions in comparison to that at WIT 0 min.

### Multiplex cytokine array

We used the Rat Cytokine 27-Plex Discovery Assay (Eve Technologies, Calgary, AB, Canada) to perform cytokine and chemokine profiling on sera from perfusate samples. The assay detected 27 different markers, including Eotaxin (CCL11), Epidermal Growth Factor (EGF), Fractalkine (CX3CL1), Interferon-γ (IFN-γ), Interleukin-1α) (IL-1α), Interleukin-1beta (IL-1β), Interleukin-2 (IL-2), Interleukin-4 (IL-4), Interleukin-5 (IL-5), Interleukin-6 (IL-6), Interleukin-10 (IL-10), Interleukin-12 (IL-12), Interleukin-13 (IL-13), Interleukin-17A (IL-17A), Interleukin-18 (IL-18), Interferon-gamma-inducible protein 10 (IP-10/CXCL10), Growth-Related Oncogene/Keratinocyte Chemoattractant (GRO/KC/CXCL1), TNF-α, Granulocyte Colony-Stimulating Factor (G-CSF), Granulocyte-Macrophage Colony-Stimulating Factor (GM-CSF), Monocyte Chemoattractant Protein-1 (MCP-1/CCL2), Leptin, Lipopolysaccharide-Induced CXC Chemokine (LIX/CXCL5), Macrophage Inflammatory Protein-1α (MIP-1α/CCL3), Macrophage Inflammatory Protein-2 (MIP-2/CXCL2), Regulated on Activation, Normal T-cell Expressed and Secreted (RANTES/CCL5), and Vascular Endothelial Growth Factor (VEGF).

### Liver injury profiling

To evaluate the extent of liver injury, we measured the levels of perfusate aspartate aminotransferase (AST), α-glutathione S-transferase (α-GST), and arginase1 (ARG1) at the end of the experiment using the Rat Liver Injury 5-Plex Featured Assay (Eve Technologies, Calgary, AB, Canada). AST is a marker of hepatocyte injury, commonly used in conjunction with other markers. α-GST is a specific marker for hepatocellular damage, particularly effective in early detection (32). ARG1 is a marker of alternative activated (anti-inflammatory) macrophage (33, 34).

### Statistical analysis

The data were presented as the median with interquartile ranges. *P* < 0.05 was considered statistically significant. The relationship between two variables was assessed using simple linear regression, and the *P* and *R*^2^ values were calculated using Prism 9 for Windows (GraphPad Software, Inc., San Diego, CA).

## Results

### Increasing WIT corresponds to higher perfusate injury markers in the *ex vivo* rat liver perfusion model

**Figure 1:**
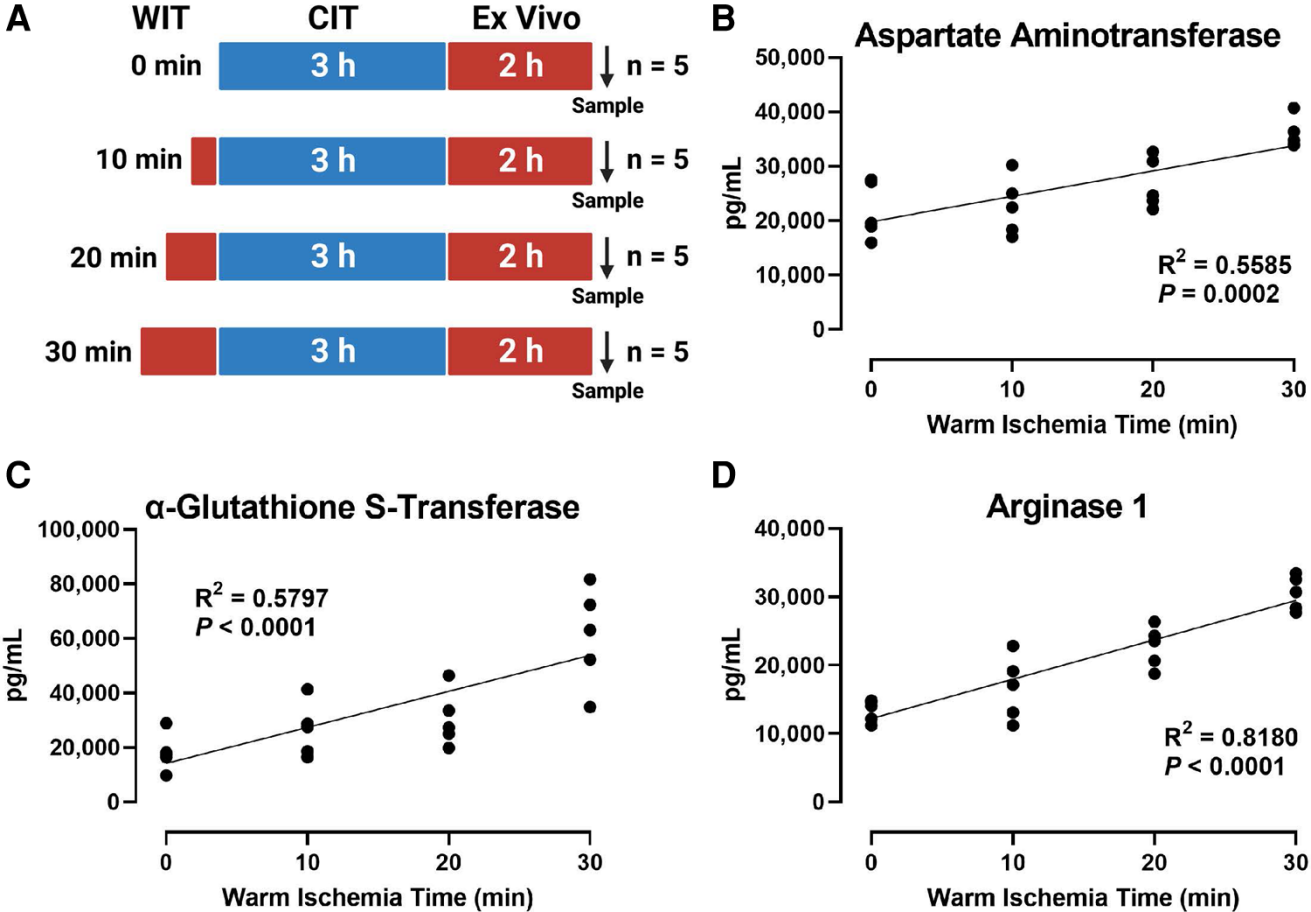
Effect of WIT on the levels of liver injury markers in the perfusate during machine perfusion. (**A**) Scheme of four experimental groups (*n* = 5 each). The rat livers underwent designated WIT of 0, 10, 20, and 30 min, followed by 3 h of cold ischemia time and 2 h of normothermic machine perfusion. Perfusate levels of (**B**) aspartate Aminotransaminase, (**C**) α-glutathione S-transferase, and (**D**) arginase1 demonstrated a significant correlation with WIT. The correlation was determined by the Spearman's rank correlation coefficient (*R*). WIT, warm ischemia time; CIT, cold ischemia time.

To induce various degrees of hepatic IRI, we exposed the rat liver to incremental periods of *in situ* WIT ranging from 0 to 30 min (10 min intervals between each group; *n* = 5 per group) before CIT and machine perfusion (Figure 1A). We evaluated liver injury by measuring perfusate markers of liver damage, such as AST, α-GST, and ARG1. We found that the duration of WIT is strongly correlated with the level of the liver injury markers AST (*R*^2^ = 0.5585, Figure 1B), α-GST (*R*^2^ = 0.5797, Figure 1C), and ARG1 (*R*^2^ = 0.8180, Figure 1D). These data suggest that WIT is an important factor in determining liver injury ensued from 3 h of CIT and 2 h of *ex vivo* machine perfusion.

### WIT differentially induces MAPK activity in the *ex vivo* rat liver machine perfusion model

**Figure 2:**
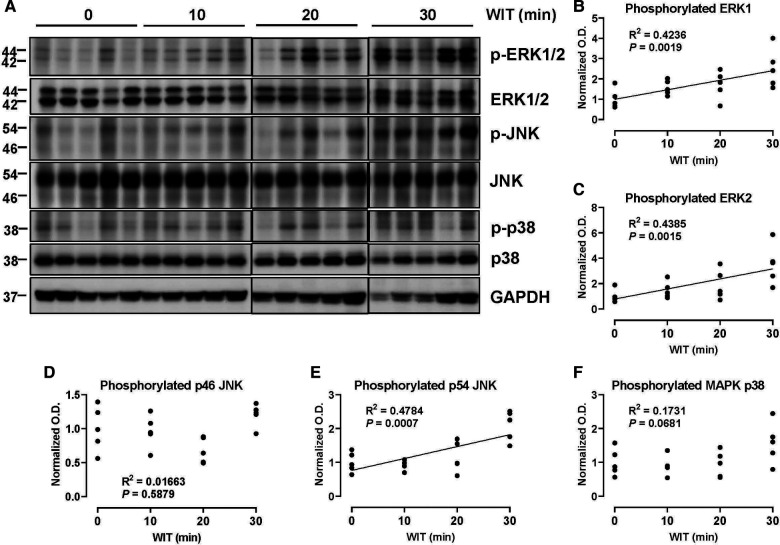
Effect of WIT on MAPK phosphorylation during machine perfusion. (**A**) Protein expression of phosphorylate ERK1/2, ERK1/2, phosphorylated p46/p54 JNK, p46/p54 JNK, phosphorylated p38 MAPK, p38 MAPK, and GAPDH. (**B–F**) The level of phosphorylated MAPKs relative to that of total MAPKs was presented by densitometry normalized to the mean value in the control group. O.D., optical density. Spearman's rank correlation coefficient (*R*) was used to determine the correlation (*n* = 20). WIT, warm ischemia time.

To determine how WIT affects MAPK activity in the *ex vivo* rat liver machine perfusion model, we analyzed the activation-loop phosphorylation of different MAPKs, a bona-fide surrogate maker for MAPK activity (35), by Western blotting of rat liver extracts collected at the end of machine perfusion. Our analysis revealed that the duration of *in situ* WIT prior to CIT and machine perfusion is strongly correlated with certain MAPK phosphorylation. Most notably, the activation-loop phosphorylation of ERK1 (Thr 202 and Tyr 204) and ERK2 (Thr 185 and Tyr 187) was substantially increased in a strong correlation with WIT (Figure 2, *R*^2^ = 0.4236 and 0.4385 for ERK1 and ERK2, respectively). Similarly, the activation-loop phosphorylation of p54 JNK (Thr 183 and Tyr 185) was also increased in a strong correlation with WIT (*R*^2^ = 0.4784). In contrast, the activation-loop phosphorylation of p46 JNK (Thr 183 and Tyr 185) and p38 MAPK (Thr 180 and Tyr 182) was not increased and did not show any correlation with WIT. These data suggest that the activity of certain MAPKs, such as ERK1/2 and p54 JNK, increase upon prolonging WIT.

### Specific cytokines decrease proportionately to the duration of WIT after machine perfusion

**Figure 3:**
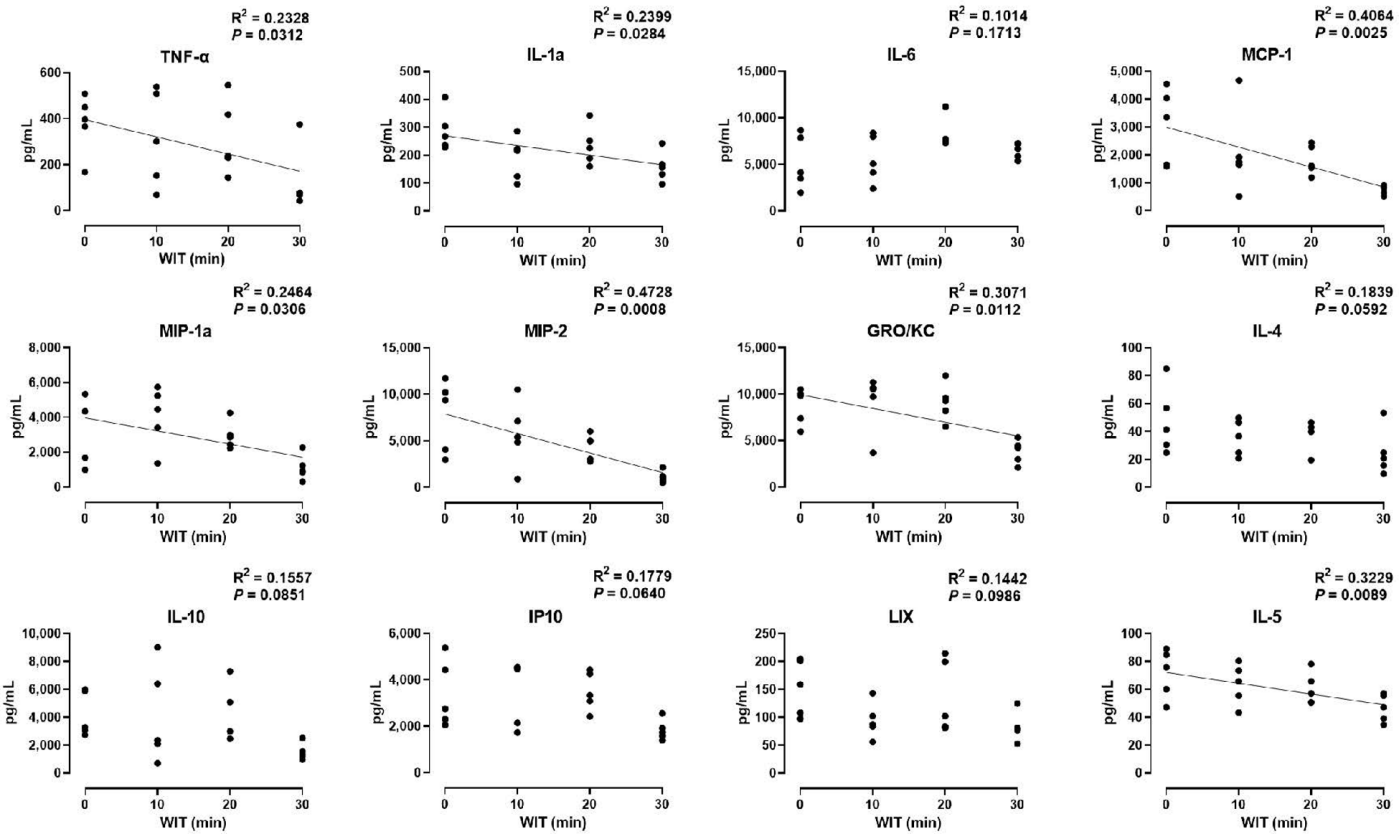
Effect of WIT on perfusate levels of cytokines in machine perfusion. Spearman's rank correlation coefficient (*R*) was used to determine the correlation (*n* = 20). A trend line is displayed only when the relationship is statistically significant. WIT, warm ischemia time.

In the *ex vivo* machine perfusion model, we examined 27 cytokine markers to find that seven of those decreased in a significant correlation with the duration of WIT, including TNF-α (*R*^2^ = 0.2328), IL-1α (*R*^2^ = 0.2399), MCP-1 (*R*^2^ = 0.4064), MIP-1α (*R*^2^ = 0.2464), MIP-2 (*R*^2^ = 0.4728), GRO/KC (*R*^2^ = 0.3071), and IL-5 (*R*^2^ = 0.3229, Figure 3). The inverse correlation between the cytokine levels and WIT suggests that prolonged WIT in the livers in *ex vivo* conditions decreases the production of these cytokines, especially MCP-1 and MIP-2. However, there was no strong correlation between WIT and IL-6, IL-4, IL-10, IP10 and LIX, suggesting that the production of these cytokines is not sensitive to WIT in the liver perfusion model.

### ERK1/2 phosphorylation correlates positively with liver injury markers but inversely with specific cytokines

**Figure 4:**
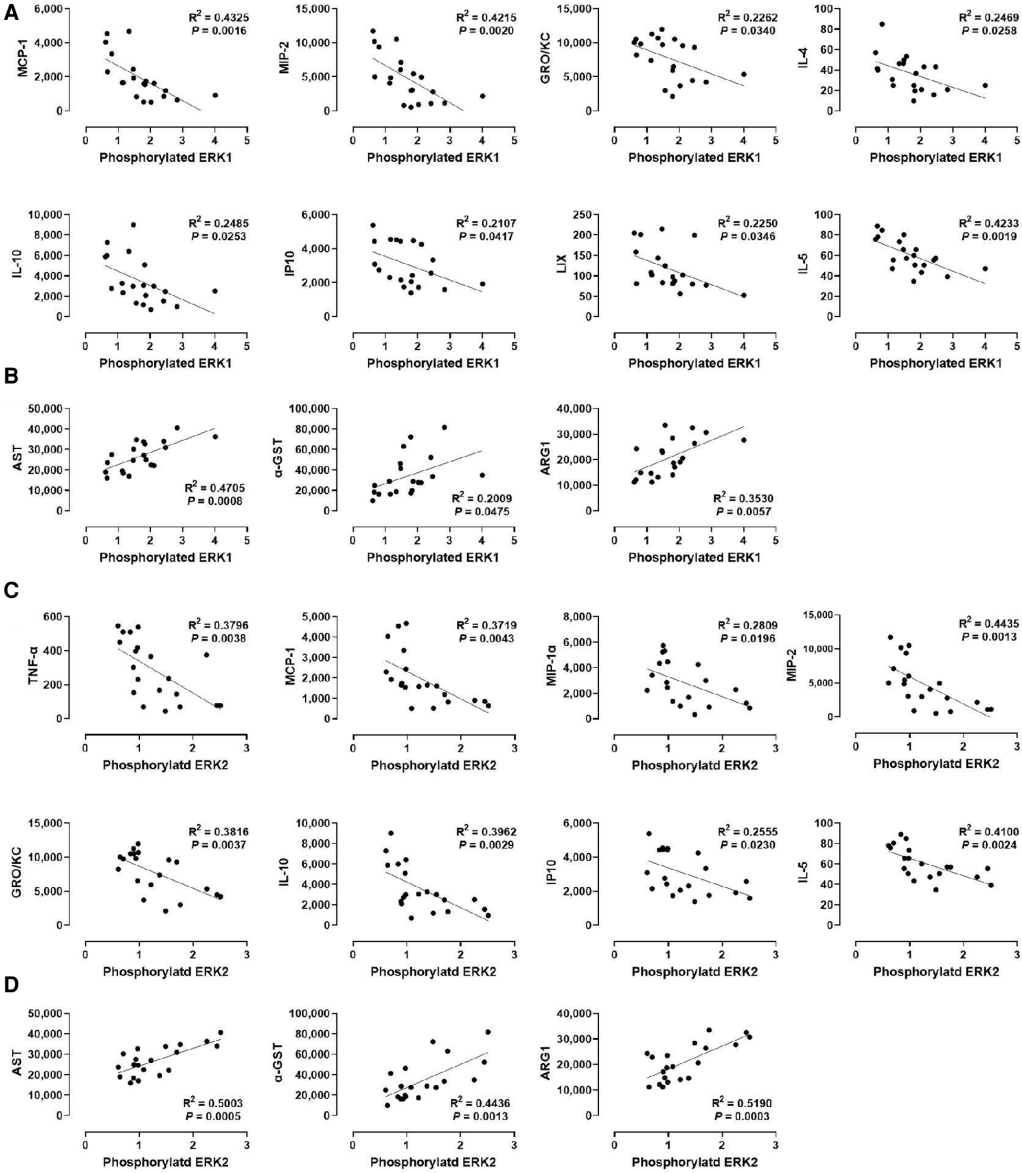
Correlation between ERK1/2 phosphorylation and perfusate levels of cytokines and liver injury markers. (**A**) ERK1 and cytokines, (**B**) ERK1 and liver injury markers, (**C**) ERK2 and cytokines, and (**D**) ERK2 and liver injury markers. Spearman's rank correlation coefficient (R) was used to determine the correlation (*n *= 20). The level of phosphorylated ERK1/2 relative to that of total ERK1/2 was presented by densitometry normalized to the mean value in the control group.

We examined the relationship between MAPK phosphorylation and perfusate levels of liver injury markers during *ex vivo* machine perfusion. Our data revealed a strong positive correlation between ERK1 phosphorylation and the liver injury markers, AST (*R*^2^ = 0.4705), α-GST (*R*^2^ = 0.02009), and ARG1 (*R*^2^ = 0.0.3530, Figure 4B). Similar as ERK1, ERK2 phosphorylation was also strongly correlated with AST (*R*^2^ = 0.5003), α-GST (*R*^2^ = 0.4436), and ARG1 (*R*^2^ = 0.0.5190, Figure 4D). These data show that ERK1/2 activity during machine perfusion may be a marker for the severity of hepatic IRI.

We next examined whether a correlation exists between ERK1/2 phosphorylation and various cytokines (Figures 4A,C). We found that the levels of MCP-1, MIP-2, GRO/KC, IL-1, IP10, and IL-5 were inversely correlated with the levels of ERK1 and ERK2 phosphorylation, at similar degrees. In contrast, the levels of IL-4 and LIX were inversely correlated only with ERK1 phosphorylation (*R*^2^ = 0.2469 and 0.2250, respectively), whereas the levels of TNF-α and MIP-1α were inversely correlated only with ERK2 phosphorylation (*R*^2^ = 0.3796 and 0.2809, respectively). These data suggest that cytokine production decreases as ERK1/2 activity increases in the liver during machine perfusion.

### P38 MAPK phosphorylation also correlates positively with liver injury markers but inversely with specific cytokines

**Figure 5:**
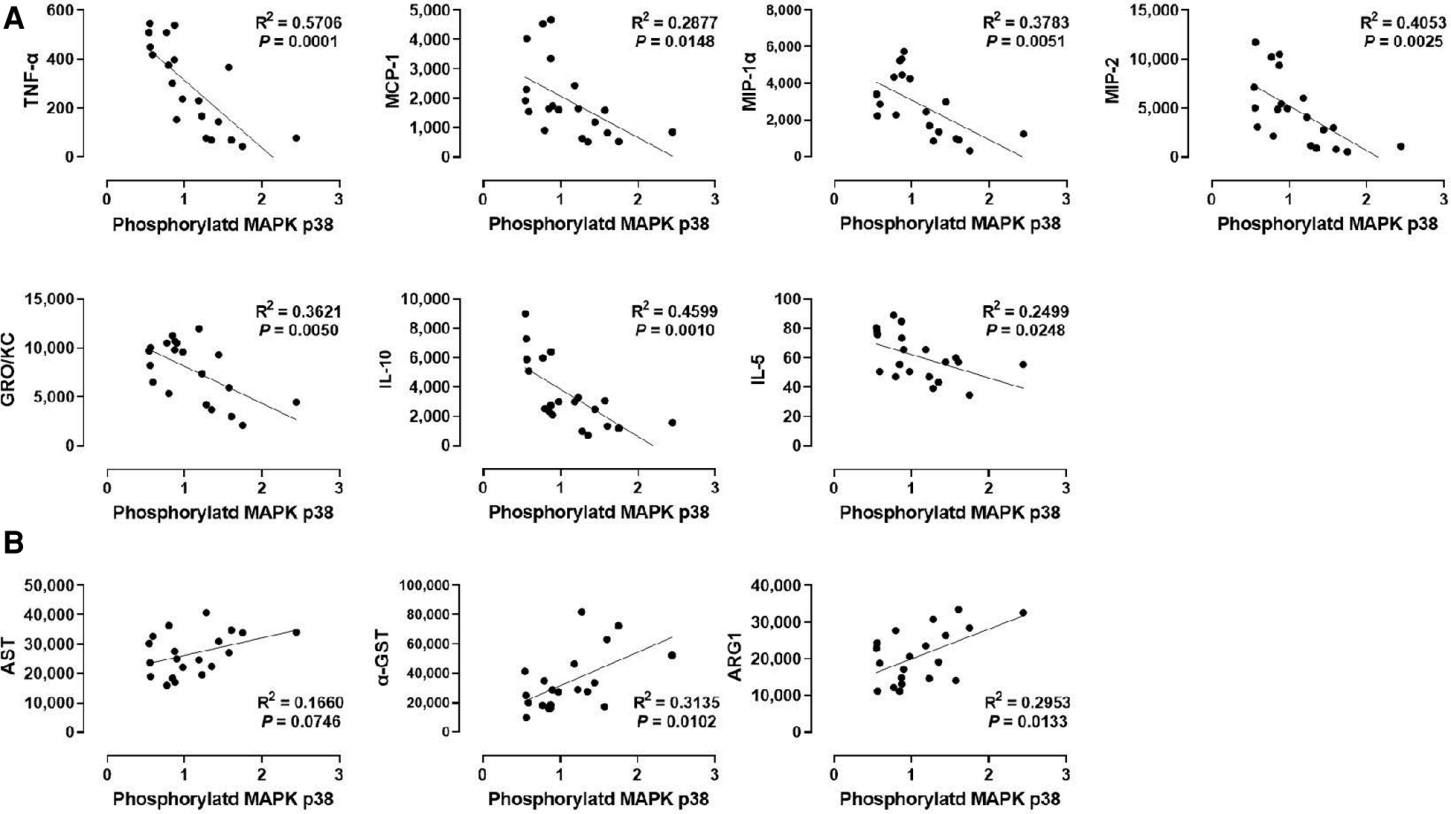
Correlation between p38 MAPK phosphorylation and perfusate levels of cytokines and liver injury markers. (**A**) p38 MAPK and cytokines and (**B**) ERK1 and liver injury markers. Spearman's rank correlation coefficient (*R*) was used to determine the correlation (*n *= 20). The level of phosphorylated p38 MAPK relative to that of total p38 MAPK was presented by densitometry normalized to the mean value in the control group.

We also analyzed the correlation between p38 MAPK phosphorylation and liver injury markers in the perfusate (Figure 5B). Although p38 MAPK did not show any correlation with WIT (Figure 2F), there was a significant positive correlation between p38 MAPK phosphorylation and the perfusate levels of α-GST (*R*^2^ = 0.3135) and ARG1 (*R*^2^ = 0.2953, Figure 5B). However, there was no significant correlation between p38 MAPK phosphorylation and AST.

Next, we examined the correlation between p38 MAPK phosphorylation levels in the liver tissues and the cytokine levels in the perfusate (Figure 5A). Like ERK1/2, p38 MAPK phosphorylation was inversely correlated with TNF-α, MCP-1, MIP-1α, MIP-2, GRO/KC, IL-1β, and IL-5. Interestingly, the correlation of these cytokines, especially TNF-α (*R*^2^ = 0.5706) and MIP-1α (*R*^2^ = 0.3783), with p38 MAPK were more like their correlation with ERK2 phosphorylation (Figure 4C) than with ERK1 phosphorylation (Figure 4A). Moreover, similar to ERK2 phosphorylation, p38 MAPK phosphorylation was not correlated with IL-4 and LIX. These data suggest that cytokine production during machine perfusion also decreases as p38 MAPK activity increases in the liver. Unlike its association with the liver injury markers and cytokine levels, p38 MAPK phosphorylation was not correlated with WIT (Figure 2F). Therefore, one may speculate that hepatic IRI, MAPK activation, and cytokine production are not in a unidirectional causal relationship.

### Phosphorylation of p54 JNK, but not p46 JNK, correlates with injury markers while various cytokines show inverse correlations with the JNK isoforms’ phosphorylation

**Figure 6:**
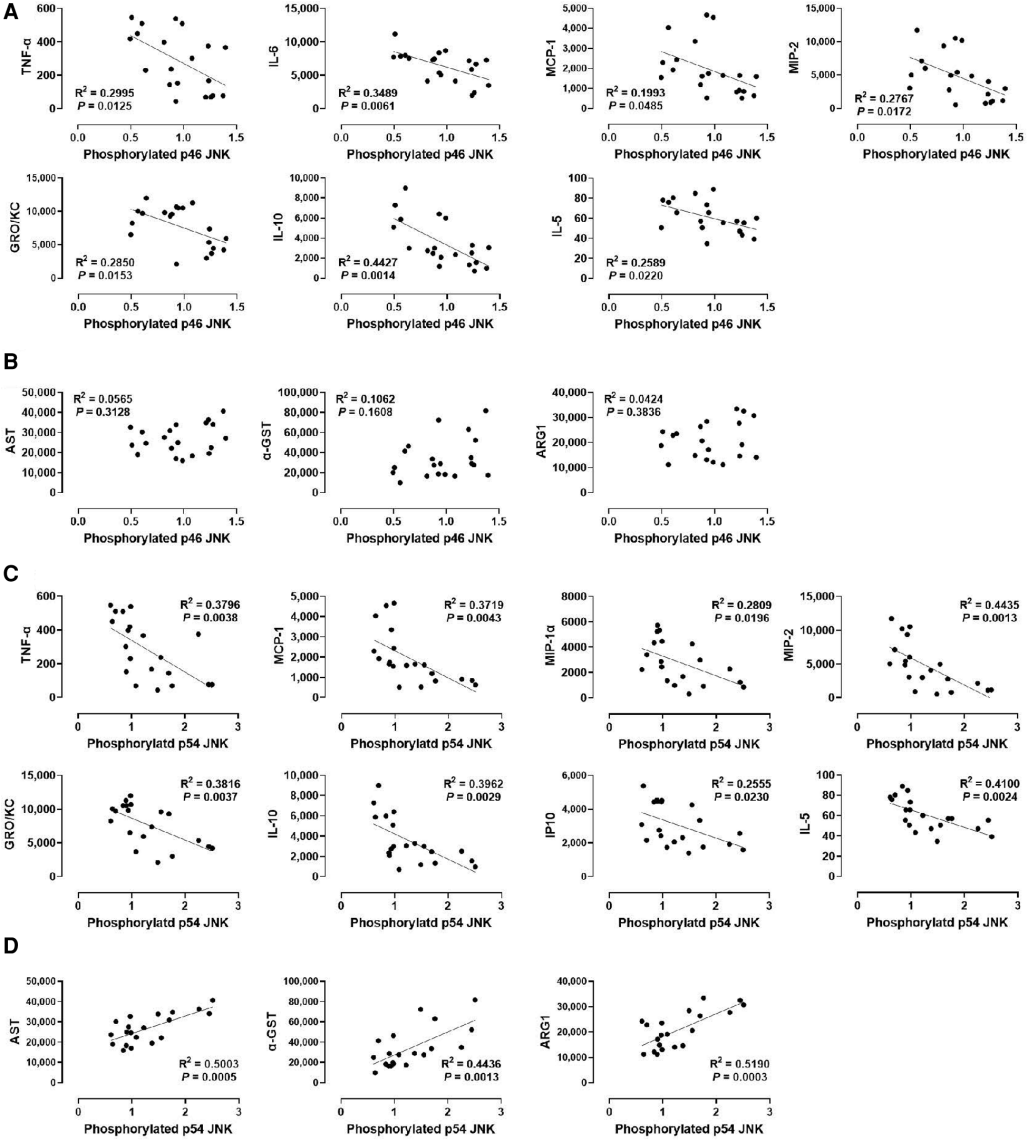
Correlation between JNK phosphorylation and perfusate levels of cytokines and liver injury markers. (**A**) p46 JNK and cytokines, (**B**) p46 JNK and liver injury markers, (**C**) p54 JNK and cytokines, and (**D**) p54 JNK and liver injury markers. Spearman's rank correlation coefficient (*R*) was used to determine the correlation (*n *= 20). A trend line is displayed only when the relationship is statistically significant. The level of phosphorylated p46/p54 JNK relative to that of total p46/p54 JNK was presented by densitometry normalized to the mean value in the control group.

Lastly, we determined whether JNK phosphorylation is correlated with injury markers and cytokine levels by examining p46 JNK and p54 JNK phosphorylation. We found that phosphorylation of p54 JNK, but not p46 JNK, was strongly correlated with all injury markers (*R*^2^ = 0.5003 for AST, *R*^2^ = 0.4436 for α-GST, and *R*^2^ = 0.5190 for ARG1; Figures 6B,D). These data suggest that p54 JNK activity, but not p46 JNK activity, correlates with the severity of hepatic IRI during machine perfusion. Interestingly, despite this differential correlation, phosphorylation of both JNK isoforms was similarly inverse-correlated with TNF-α, MCP-1, MIP-2, GRO/KC, IL-10, and IL-5 (Figures 6A,C). The only difference was that p46 JNK phosphorylation was inversely correlated with IL-6 whereas p54 JNK phosphorylation was inversely correlated with MIP-1α and IP10. These data suggest that cytokine production also decreases as JNK activity increases in the liver during machine perfusion.

## Discussion

Given the brief period of machine perfusion application for donor livers, typically only a few hours (4), it is essential to understand rapid cellular signal changes in response to injury during liver machine perfusion. MAPKs, which show an immediate stress response through phosphorylation (9, 36), serve as promising biomarkers. In an *ex vivo* rat liver machine perfusion model, this study examined the relationship between WIT, MAPK activation, and cytokine production. Our data reveal novel positive correlations between WIT and injury markers in the initial 2 h of perfusion along with relationships between WIT with ERK1/2 and p54 JNK phosphorylation levels corresponding to injury severity.

Understanding the association between MAPKs and cytokines during liver injury is important, as they can significantly influence each other's activities. In the early phase of acute liver injury, Kupffer cells release cytokines and chemokines, which are sensed by circulating immune cells (37, 38). However, it is unclear whether this pathophysiological context can be applied to *ex vivo* machine perfusion conditions. Cytokine production will be compromised during *ex vivo* machine perfusion due to the absence of bone marrow-derived monocytes (27), extrahepatic macrophages (10), or neutrophils (39) in the *ex vivo* circuit, unless the native Kupffer cell function is preserved. The decrease in perfusate cytokine levels with increasing WIT in our findings appears counterintuitive, considering that in a clinical context, prolonged WIT typically results in more pronounced injury and presumed elevated inflammation. This unexpected observation can be explained by the lack of key inflammatory contributors during machine perfusion. The impacts of different WIT durations on IRI become evident following reperfusion with host blood, which contains a complete array of blood cells, including neutrophils and platelets. These elements are crucial for triggering a comprehensive inflammatory cascade and its eventual resolution (10). The combined impact of cytokine-mediated cellular interactions on IRI, following macrophage-driven cytokine production, can be dualistic, potentially leading to either damage or restoration. This is due to the multi-faceted role of macrophages that extends beyond merely triggering inflammation; they also play a significant part in its regulation and resolution (40). A recent study using discarded human livers found that high levels of cytokine in the perfusate may indicate recovery of local immune cell function during normothermic machine perfusion (29). In our study, perfusate cytokine levels showed an inverse correlation with WIT, whereas liver injury markers showed a positive correlation with WIT. This suggests that preserving Kupffer cells can also be protective against IRI after LT. For example, Kupffer cell depletion resulted in increased susceptibility to hepatic IRI, whereas ablation of circulating monocytes did not (41). Furthermore, Kupffer cells can be categorized into immunoregulatory and pro-inflammatory subsets based on gene signature (38, 42). Kupffer cell-originated HO-1 mediates the protected phenotype (43), and switches macrophages from classical (pro-inflammatory) to alternate (anti-inflammatory) activation via MAPK activation (44).

Our study found a significant inverse correlation between the activation-phosphorylation of MAPKs and levels of cytokines modulating immune cell behavior (i.e., MCP-1, MIP-2, GRO/KC, IL-10, and IL-5; Figures 4–6) in the perfusate. The relationship between cytokines and MAPKs during hepatic IRI is complex and involves various interacting factors. For example, cytokines can activate JNK and p38, which can subsequently induce pro-inflammatory cytokine production (45). In contrast, ERK1/2 activation is associated with anti-inflammatory cytokines such as IL-10 production and decreased IL-12 production (46–49). Additionally, MAPKs can impact each other, such as phosphorylated p38 inhibiting ERK1/2 during hepatic IRI (50). MAPK activation can also directly affect intracellular signals of hepatocytes during hepatic IRI, such as JNK's association with the endoplasmic reticulum (ER) stress response, the mitochondrial pathway of apoptosis, and autophagy (51). Moreover, MAPK isoforms may have distinct roles during hepatic IRI. For instance, TNF-α preferentially activates isoforms within the p46 JNK subfamily during macrophage regulation (52), while specific isoforms of ERK1 and ERK2 are responsible for determining the survival and proliferation of hepatocytes, respectively (52). Our data also suggests that roles of ERK1 and ERK2 may not be entirely redundant during hepatic IRI. For example, given the similarity between ERK2 and p38 MAPK in our results, p38 MAPK function may be more closely associated with ERK2 than with ERK1 during machine perfusion. Therefore, the relationship between MAPK activation and cytokines can be direct and indirectly mediated via other MAPK, different isoforms of MAPK, and extra-cellular interactions. Decoding cell signals associated with cytokine interaction in hepatic IRI will require further mechanistic studies based on the specific kinase inhibitors (53–56). Such knowledge will aid in the development of advanced organ resuscitation methods such as a combination targeted therapy in the MAPK pathway during machine perfusion.

Interpretation of the results warrants certain considerations. Factors such as the choice of preservation and machine perfusion conditions, including the initial cold liver flush [using lactated Ringer's solution (57, 58)] and the components of the perfusate (as detailed in Table 1), could potentially impact the study outcomes. In this study, only male rats were utilized to obtain sufficiently large size of livers for our machine perfusion system from the age-matched rats. Consequently, further investigations under varying conditions may be necessary to substantiate our findings. Additionally, the intrinsic complexities of *ex vivo* liver machine perfusion complicate the establishment of controls because obtaining perfusate samples from a liver without ischemic time is impracticable. In response to these challenges, we derived a linear correlation from outcomes across four incrementally longer WIT durations in addition to the duration of CIT and machine perfusion, providing an alternative assessment of specific factors’ influences.

In summary, our results revealed a positive relationship between WIT and perfusate injury markers, linked to ERK1/2 and p54 JNK phosphorylation in an *ex vivo* rat liver machine perfusion model. Moreover, an inverse correlation was observed between MAPK phosphorylation and perfusate levels of cytokines modulating immune cell behavior. These findings indicate that MAPK activation is an early response during machine perfusion and strongly correlates to the pre-existing injury level of the liver. To further determine the role of MAPK activation on cytokine production during hepatic IRI, a study design that specifically regulates each MAPK would be necessary, and further comprehensive research is needed to translate the utility of MAPK regulation in hepatic IRI from the laboratory to clinical application, which may provide an opportunity to development of a novel pharmacological strategy during machine perfusion.

## Data Availability

The raw data supporting the conclusions of this article will be made available by the authors, without undue reservation.

## Supplementary material

Supplementary Figure 1 Immunoblotting analysis of liver tissue lysates. Protein expression of phosphorylated ERK1/2, ERK1/2, phosphorylated p46/p54 JNK, p46/p54 JNK, phosphorylated p38 MAPK, p38 MAPK, and GAPDH were examined in each group. Samples from each WIT group and the control group (0 min) were run together to compare the differences. Glyceraldehyde-3-phosphate dehydrogenase (GAPDH) was used as a loading control.
