# Supplementary figures and images for: Prolonged warm ischemia time increases mitogen-activated protein kinase activity and decreases perfusate cytokine levels in ex vivo rat liver machine perfusion
Source: Front Transplant. 2023 Aug 25;2:1215182. doi: 10.3389/frtra.2023.1215182 (PMC11235240; doi:10.3389/frtra.2023.1215182)

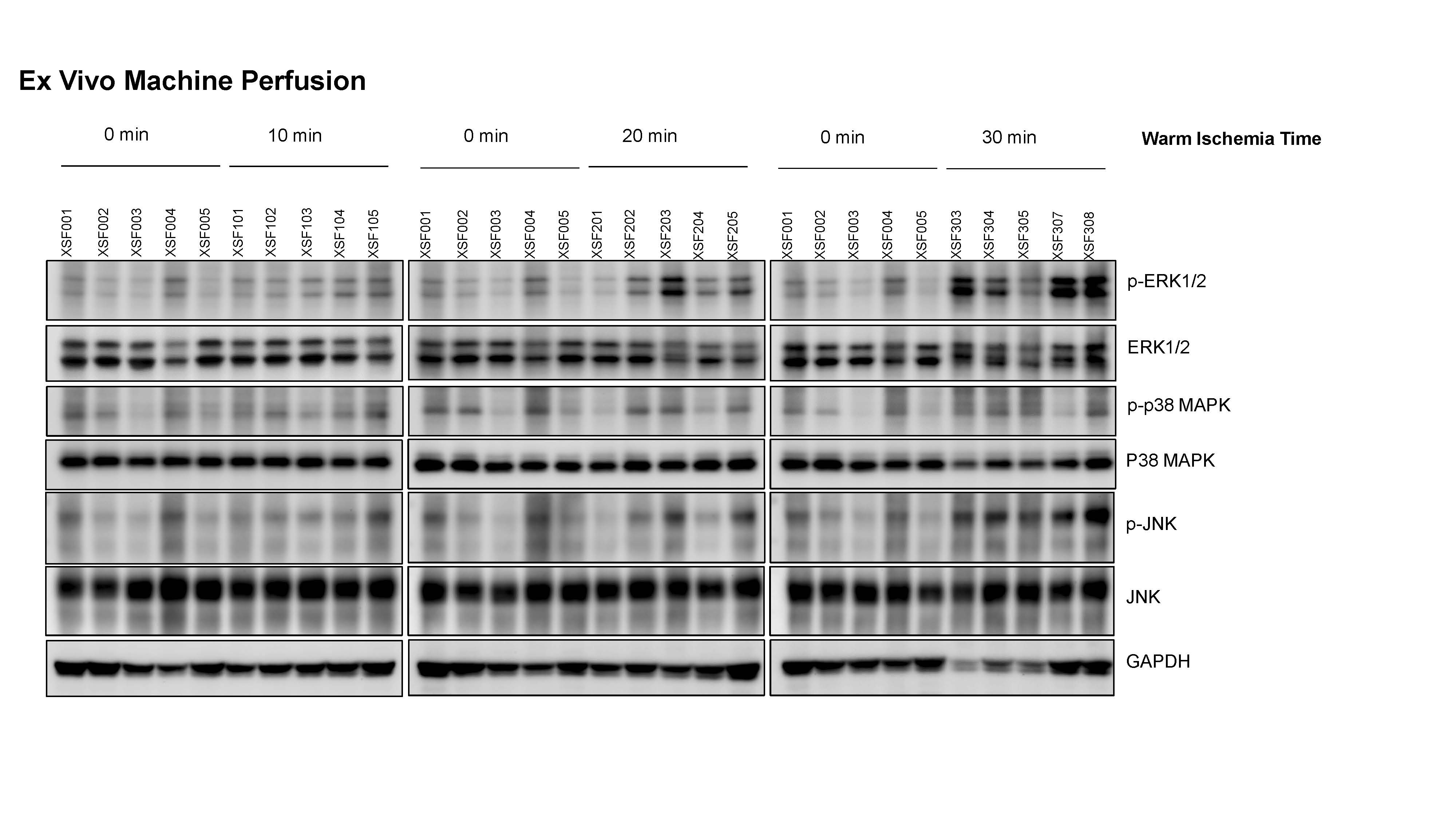

Supplement: Supplementary Figure 1 — Immunoblotting analysis of liver tissue lysates. Protein expression of phosphorylated ERK1/2, ERK1/2, phosphorylated p46/p54 JNK, p46/p54 JNK, phosphorylated p38 MAPK, p38 MAPK, and GAPDH were examined in each group. Samples from each WIT group and the control group (0 min) were run together to compare the differences. Glyceraldehyde-3-phosphate dehydrogenase (GAPDH) was used as a loading control. [file Image1.jpeg]
